# Static Magnetic Fields Modulate the Response of Different Oxidative Stress Markers in a Restraint Stress Model Animal

**DOI:** 10.1155/2018/3960408

**Published:** 2018-05-14

**Authors:** E. Coballase-Urrutia, L. Navarro, J. L. Ortiz, L. Verdugo-Díaz, J. M. Gallardo, Maria Eugenia Hernández, F. Estrada-Rojo

**Affiliations:** ^1^Laboratory of Neuroscience, National Institute of Pediatrics, 04539 Ciudad de México, Mexico; ^2^Physiology Department, Faculty of Medicine, UNAM, 04150 Ciudad de México, Mexico; ^3^Tominaga Nakamoto School of Medicine, 53390 Naucalpan de Juarez, MEX, Mexico; ^4^Laboratory of Nephrology Specialties Hospital, XXI Century National Medical Center, IMSS, 06720 Ciudad de México, Mexico; ^5^Laboratory of Neuropharmacology, Subdirection of Clinical Investigations, National Institute of Psychiatry "Ramón de la Fuente Muñiz", 14370 Ciudad de México, Mexico

## Abstract

Stress is a state of vulnerable homeostasis that alters the physiological and behavioral responses. Stress induces oxidative damage in several organs including the brain, liver, kidney, stomach, and heart. Preliminary findings suggested that the magnetic stimulation could accelerate the healing processes and has been an effective complementary therapy in different pathologies. However, the mechanism of action of static magnetic fields (SMFs) is not well understood. In this study, we demonstrated the effects of static magnetic fields (0.8 mT) in a restraint stressed animal model, focusing on changes in different markers of oxidative damage. A significant increase in the plasma levels of nitric oxide (NO), malondialdehyde (MDA), and advanced oxidation protein products (AOPP), and a decrease in superoxide dismutase (SOD), glutathione (GSH), and glycation end products (AGEs) were observed in restraint stress model. Exposure to SMFs over 5 days (30, 60, and 240 min/day) caused a decrease in the NO, MDA, AGEs, and AOPP levels; in contrast, the SOD and GSH levels increased. The response to SMFs was time-dependent. Thus, we proposed that exposure to weak-intensity SMFs could offer a complementary therapy by attenuating oxidative stress. Our results provided a new perspective in health studies, particularly in the context of oxidative stress.

## 1. Introduction

Stress is a state of vulnerable homeostasis that alters the physiological and behavioral responses. The alterations depend on the severity of stress, as well as the type and duration of the stressful events. Attempts to reestablish the homeostasis of the body result in the dysfunction of the central nervous system (CNS) [1–3] and systemic dysfunction [4]. CNS alterations may be accountable for the development of psychiatric disorders, such as depression, which are associated with cognitive alterations [5]. In addition, major changes in hemodynamic parameters such as heart rate and arterial blood pressure have also been reported [6, 7]. Furthermore, it has been shown that stress induces oxidative damage in the brain [8], liver [9], and kidney [10], gastric lesions [11], hyperlipidemia (implicated in the etiology of atherosclerosis), and cardiovascular problems in different animal models [12].

Recent studies have suggested that psychological or physiological stress is associated with oxidative stress [8, 13]. Oxidative stress has also been shown to be a main factor in the progression of many disorders such as neurodegenerative diseases [14], chronic kidney disease [15], liver inflammation [16], hypercholesterolemia [17], and diabetes [18]. Thus, the morbidity rate of various disorders is increased upon long-term exposure to physiological and psychological stress [19].

A well-known method for inducing physiological and physical stress [20] and for mimicking the natural progression from acute to chronic stress is the immobilization stress or restraint stress model [19, 21].

The effects of static magnetic fields (SMFs) have been evaluated in cells, cellular components, genetic material, embryogenesis, and the CNS. These studies demonstrated that exposure to SMF could have both beneficial and detrimental biological consequences. For example, SMF exposure increases the total antioxidant capacity, decreases allergic inflammation [22], enriches trace elements [23], and modulates DNA damage and/or damage repair, possibly through mitochondrial mechanisms [24, 25]. SMFs could also inhibit the proliferation of some human cells [26], increase apoptosis and necrosis via changes in the cell viability and lipid peroxidation [27], and stimulate lipid peroxidation in the liver [28]. However, there is currently no definitive evidence of health risk from exposure to SMFs.

Although the mechanism of action of SMFs is not well understood, preliminary findings from* in vivo *and* in vitro *clinical studies have suggested that magnetic stimulation could accelerate the healing process and provide an effective complementary therapy for different pathologies [29–32]. It may also induce changes in living systems, at the organism, tissue, cellular, membrane, and subcellular levels.

In the current study, we investigated the possible impact of SMFs on an animal model of restraint stress, focusing on different plasmatic markers of oxidative damage.

## 2. Materials and Methods

### 2.1. Experimental Animals

Experiments were performed on 56 Wistar rats (200–250 g) obtained from the animal breeding center of the Faculty of Medicine in the University City, UNAM. The animal center maintained constant humidity of 60%, temperature of 22°C, and air ventilation, with a 12 h light-dark cycle. The experimental protocols were approved by the local committee of research and ethics of UNAM Faculty of Medicine (088/2013).

**Figure 1:**
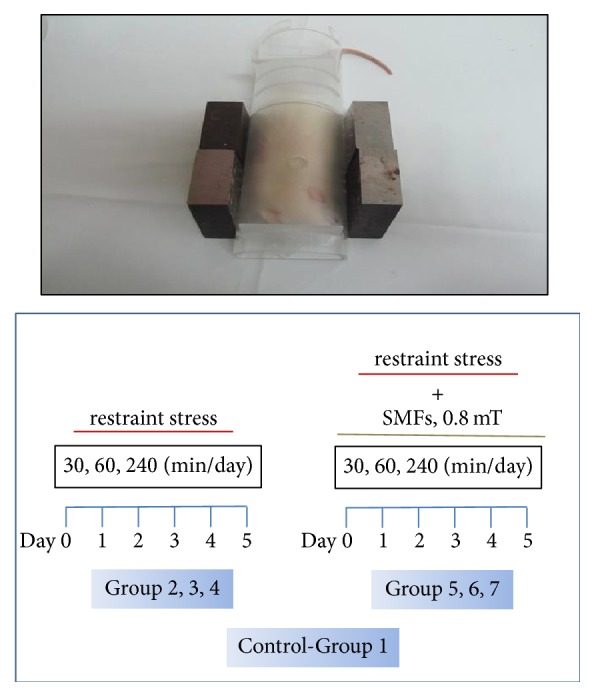
SMF stimulations. The rat was placed in a transparent acrylic box (8 cm wide and 25 cm long) and the ferromagnetite magnets were placed at both sides of the box.

The experimental animals were separated into seven groups of eight rats each. Group 1 consisted of the control group. Groups 2, 3, and 4 were maintained in transparent acrylic restraining boxes for 30, 60, and 240 min, respectively. Groups 5, 6, and 7 were also restrained in the same type of restraining boxes for 30, 60, and 240 min, respectively, but during these periods, magnetic fields were applied by placing iron alloy magnets in the boxes. Thus, a constant magnetic field of 0.8 mT was produced. The rats were placed in the restraining boxes at the same times each day. The experiment lasted for 5 days (Figure 1).

### 2.2. SMF Stimulation System

During the stimulation, each rat was placed in a transparent acrylic box (8 cm wide and 25 cm long). The ferromagnetite magnets were placed at both sides of the box. The intensity of the magnetic field was 0.8 mT, which was measured with a gasometer over 1 year. The magnets were placed in a north-south position.

### 2.3. Reagents

The salts, reagents, and the markers for the molecular weights (MW) were obtained from Sigma-Aldrich (CA, USA), the chromatography columns from Bio Rad (Hercules, CA, USA), and the plates from Becton Dickinson (Labwere, Lincoln Park, NJ, USA).

### 2.4. Sample Collection

The rats were sacrificed by decapitation, and the blood was collected in tubes containing ethylenediaminetetraacetic acid (EDTA) and centrifuged at 1300*g* to separate the plasma. The blood samples were subsequently frozen at −20°C and further subjected to assays for nitric oxide (NO), superoxide dismutase (SOD), glutathione (GSH), glycation end products (AGEs), advanced oxidation protein products (AOPP), and thiobarbituric acid reactive substances (TBARS).

### 2.5. Measurement of Total Protein

This was performed by using the Bradford method, with Coomassie Blue as the dye. The standard calibration curve for albumin fraction V was used [33].

### 2.6. Oxidative Damage Assays

#### 2.6.1. NO Assay

The plasma nitrate and nitrite concentrations were determined using the Griess reaction. Briefly, plasma was incubated with an equal volume of nitrate reductase in 0.1 M potassium phosphate buffer, containing 1 mM *β*-nicotinamide adenine dinucleotide phosphate (NADPH) and two units of nitrate reductase per milliliter. Samples were incubated overnight at 37°C. Griess reagents (1% sulphanilamide and 0.1% naphthyl-ethylene diamine-dihydrochloride in 5% phosphoric acid) were added, and the samples were incubated for an additional 115 min at room temperature. The total amount of nitrite was measured at 540 nm and expressed in terms of *μ*mol/L [34].

#### 2.6.2. SOD Assay

The SOD activity was assayed using a previously reported method [35]. A competitive inhibition assay was performed using the xanthine-xanthine oxidase system to reduce nitroblue tetrazolium (NBT). The reaction mixture had a final volume of 166 *μ*L, comprising 0.122 mM EDTA, 30.6 *μ*M NBT, 0.122 mM xanthine, 0.006% bovine serum albumin, and 49 mM sodium carbonate. A specific homogenate (33 *μ*L; 1 : 50 dilution) was added to the reaction mixture, followed by 30 *μ*L of a xanthine oxidase solution to obtain a final concentration of 2.5 U/L; this mixture was incubated at room temperature for 30 min. The reaction was terminated by the addition of 66 *μ*L of 0.8 mM cupric chloride, and the optical density was measured at 560 nm. An NBT reduction of 100% was achieved in a tube in which the sample was replaced with distilled water. The amount of protein that inhibited 50% of NBT reduction was defined as having one unit of SOD activity. The results were expressed in terms of Umol/mg/protein.

#### 2.6.3. GSH Assay

The GSH assay was based on the reaction between GSH and 5,5′-dithio-bis-(2-nitrobenzoic acid) (DTNB), which produces the TNB chromophore, with maximal absorbance at 412 nm, and the oxidized glutathione–TNB adduct (GS–TNB). The rate of formation of TNB, measured at 412 nm, was proportional to the concentration of GSH in the sample. The disulfide product (GS–TNB) was then reduced by GR in the presence of NADPH, recycling GSH back into the reaction. The rate of change measured at an absorbance of 412 nm was found to be linearly proportional to the total concentration of GSH. The plasma was diluted with the KPE buffer (0.1 M potassium phosphate, 5 mM disodium EDTA, pH 7.5), prior to the addition of DNTB (2.5 mM) and GSH reductase solutions (250 U/mL). Following the addition of *β*-NADPH, the absorbance (420 nm) was measured immediately at 30 s intervals for 2 min. The rate of change in absorbance (U/mg/protein) was compared to that observed in the GSH standards [36].

#### 2.6.4. Glycation Assay

To measure the AGE formation, the samples were incubated for 6 weeks at 37°C in the dark and then dialyzed (48 h in phosphate-buffered saline [PBS], pH 7.4, with one change of dialysis solution). The characteristic fluorescence of total AGE, as well as its high- (HMW-AGE) and low-molecular-weight fractions (LMW-AGE) after their separation according to the method described [37], was measured with a spectrofluorometer with 370 and 440 nm as the excitation and emission wavelengths, respectively. The mean values measured were presented in terms of arbitrary fluorescence units (AFU).

#### 2.6.5. AOPP Assay

The concentration of total AOPP, as well as that of its high- and low-molecular weight fractions, was measured according to a previously described method [38]. The separation of LMW- and HMW-AOPP was performed in conditions similar to that for AGE separation [39]. To measure the AOPP formation, the samples were incubated for 60 min at 37°C in the dark and then dialyzed (48 h in PBS, pH 7.4, with one change of dialysis solution). The characteristic color was measured at 340 nm. The standard curve (made with chloramine T with concentrations of 0–100 *μ*M) was used to calculate the AOPP concentration (*μ*M).

### 2.7. Estimation of Lipid Peroxidation

The malondialdehyde (MDA) content in the whole tissue homogenate was estimated using a standard curve of trimethoxypropane. The reaction mixture consisted of 0.026 M TBA, 0.211 M hypochloric acid, 6.66% trichloroacetic acid, and 1 mM desferrioxamine B. Each tissue homogenate (200 *μ*L) was added to 1000 *μ*L of the reaction mixture, vortexed vigorously, and then heated at 100°C for 10 min. The mixture was cooled down and 1 mL of n-butanol-pyridine (15 : 1) was added. After centrifugation at 1,200 ×g for 10 min, the organic layer was separated and the absorbance was measured at 530 nm. MDA is an end-product of lipid peroxidation that reacts with TBA and results in TBARS, which is expressed in terms of *μ*moles/MDA/L [40].

### 2.8. Statistical Analysis

Data were expressed as the mean ± standard error of the mean (SEM) and analyzed by one-way analysis of variance (ANOVA), followed by a Dunnett's multiple comparison test. Statistical significance was assumed at a *p* < 0.05.

## 3. Results

### 3.1. NO Assay

**Figure 2:**
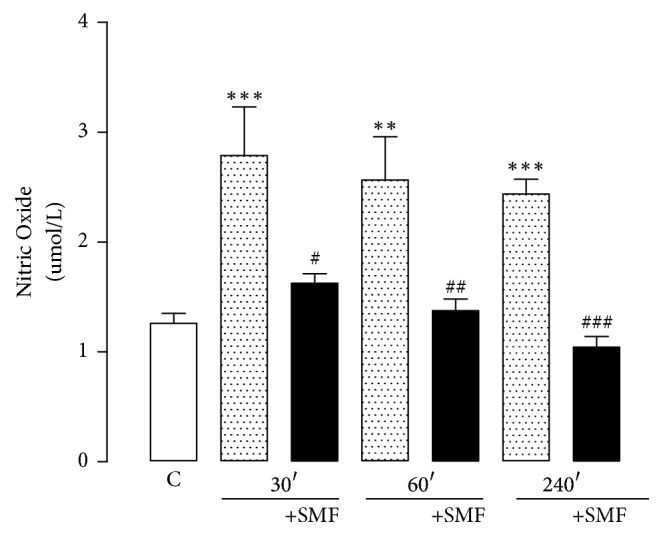
Effect of restraint stress on the level of nitric oxide with and without static magnetic field (SMF, 0.8 mT) for 30, 60, and 240 min. The results were analyzed by one-way analysis of variance (ANOVA), and Dunnett's multiple comparison test was used to compare the outcomes of the experimental and control groups. Each determination was performed in duplicate, and data are expressed as mean ± standard error of the mean (*n* = 8 per group). ^*∗∗∗*^*p* < 0.001 versus control group; ^*∗∗*^*p* < 0.01 versus control group, ^#^*p* < 0.05 versus 30 min without SMF; ^##^*p* < 0.01 versus 60 without SMF, and ^###^*p* < 0.001 versus 240 min without SMF.

Restraint stress induced a significant increase in the plasma NO levels in each time of restriction (30 min, 121%; 60 min, 103%, and 240 min, 93%). SMF stimulation decreased the NO levels in a time-dependent manner as compared with the groups that did not receive SMFs (30 min, 58%; 60 min, 54%, and 240 min, 43%; Figure 2).

### 3.2. SOD Assay

**Figure 3:**
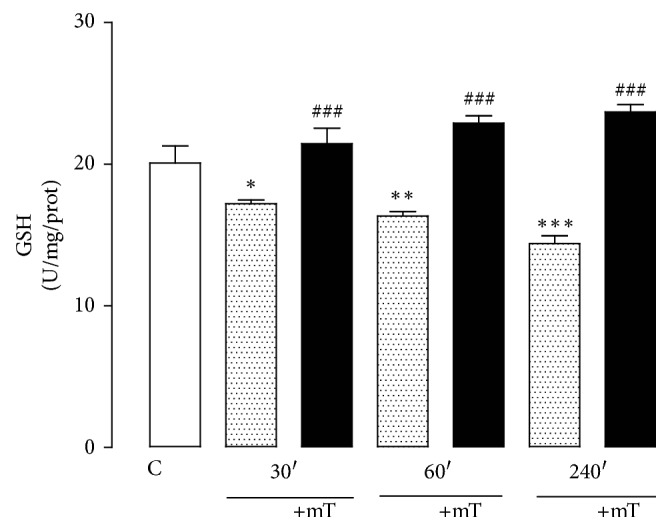
Effect of restraint stress on the GSH activity with and without SMF (0.8 mT) during 30, 60, and 240 min. The analysis of variance (ANOVA) and Dunnett's multiple comparison test were used to compare outcomes between experimental and control groups. Data are represented by the mean ± standard error of the mean (*n* = 8 per group). ^*∗*^*p* < 0.05; ^*∗∗*^*p* < 0.01, and ^*∗∗∗*^*p* < 0.001 versus control group; ^###^*p* < 0.001 versus 30, 60, and 240 min without SMF.

With restraint stress treatment, the activity of SOD decreased significantly in all groups (30 min, 22%; 60 min, 20%; and 240 min, 20%). The effect of exposure to SMF was similar to that exhibited by the control group. Additionally, we observed an increase in the SOD activity at both 60 min (29%) and 240 min (32%). The results indicated that exposure to SMFs decreased the oxidant activity caused by O_2_^∙^ (Figure 3).

### 3.3. GSH Assay

**Figure 4:**
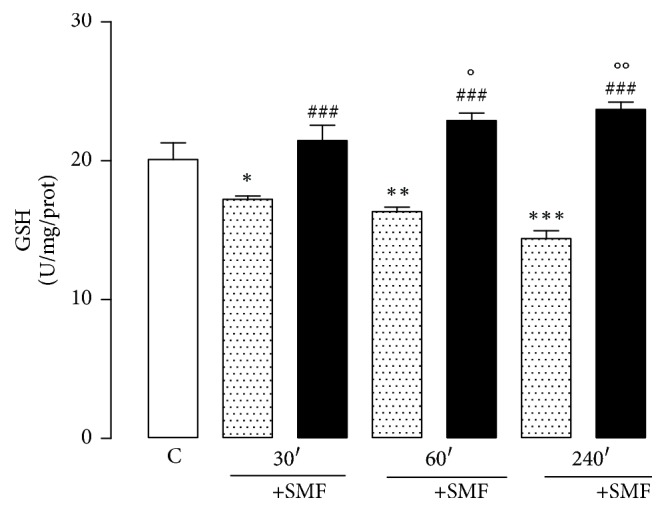
Effect of restraint stress on the GSH activity with and without SMF (0.8 mT) during 30, 60, and 240 min. The analysis of variance (ANOVA) and Dunnett's multiple comparison test was used to compare outcomes between experimental and control groups. Data are represented by the mean ± standard error of the mean (*n* = 8 per group). ^*∗*^*p* < 0.05; ^*∗∗*^*p* < 0.01, and ^*∗∗∗*^*p* < 0.001 versus control group; ^###^*p* < 0.001 versus 30, 60, and 240 min without SMF; °*p* < 0.05 and °°*p* < 0.01 versus control group.

The level of plasma GSH significantly decreased in all restrained groups compared with the control group (30 min, 14%; 60 min, 18%; 240 min, 28%). However, the groups that were exposed to SMF showed a significant increase in plasma GSH levels (30 min, 24%; 60 min, 40%; 240 min, 64%). We additionally observed a significant increase in the 60 (14%) and 240 (17%) min SMF groups compared with the control group (Figure 4).

### 3.4. Glycation End Products (AGEs)

**Figure 5:**
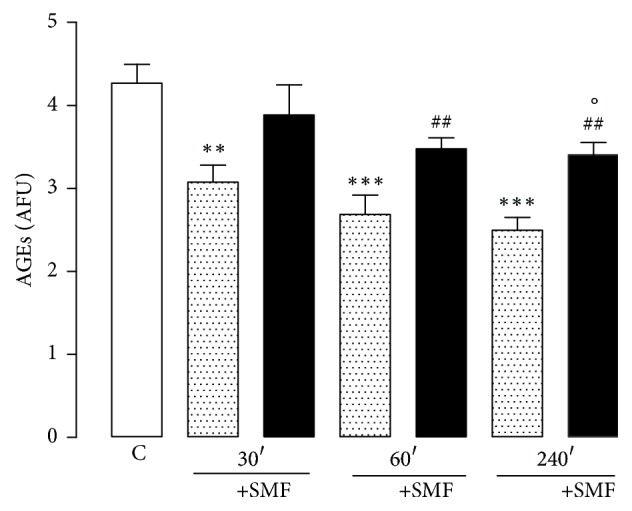
Effect of restraint stress on the levels of AGEs with and without SMF (0.8 mT) during 30, 60, and 240 min. The results were analyzed by analysis of variance (ANOVA), and Dunnett's multiple comparison test was used to compare the outcomes between the experimental and control groups. Data are represented by the mean ± standard error of the mean (*n* = 8 per group). ^*∗∗*^*p* < 0.01 and ^*∗∗∗*^*p* < 0.001 versus control group; ^##^*p* < 0.01 versus 60 and 240 min without SMF and °*p* < 0.05 versus control group.

The plasma AGEs levels were significantly lower in the restraint groups compared with the control group (30 min, 28%; 60 min, 37%; 240 min, 42%). However, the groups that were treated with SMF showed an increase in plasma AGE levels compared with the restraint groups (30 min, 26%; 60 min, 29%; and 240 min, 36%). SMF treatment for 240 min resulted in a significant decrease compared with the control group (Figure 5).

### 3.5. Assay of Oxidation Protein Products

**Figure 6:**
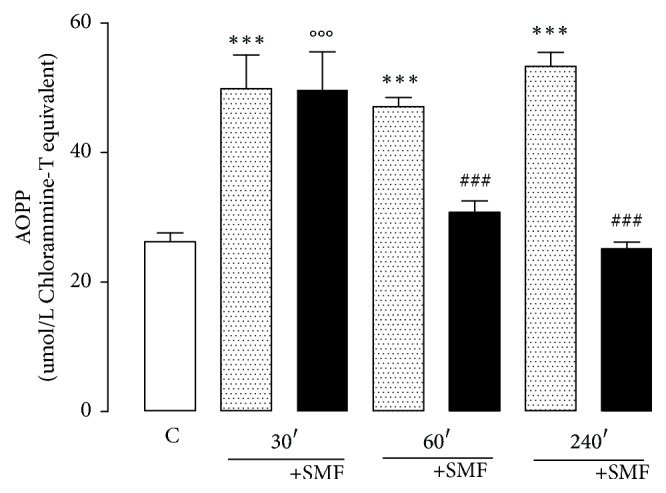
Effect of restraint stress on the AOPP levels with and without SMF (0.8 mT) during 30, 60, and 240 min. The results were analyzed by analysis of variance (ANOVA), and Dunnett's multiple comparison test was used to compare the outcomes between experimental and control group. Data show the mean ± standard error of the mean (*n* = 8 per group). ^*∗∗∗*^*p* < 0.001 versus control group; ^###^*p* < 0.001 versus 60 and 240 min without SMF; °°°*p* < 0.001 versus control group.

A significant increase in the level of AOPP was observed after the restraint stress in all experimental groups without SMF (30 min, 90%; 60 min, 79%; 240 min, 103%). Exposure to SMF for 60 and 240 min, but not for 30 min, significantly decreased the AOPP levels (35% and 53%, resp.), indicating an antioxidant effect of SMF (Figure 6).

### 3.6. MDA Assays

**Figure 7:**
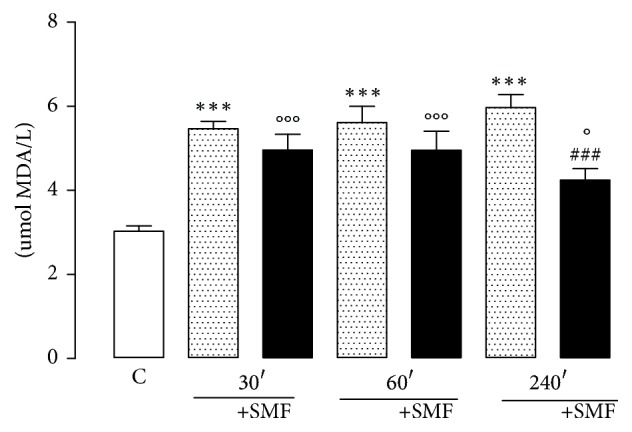
Effect of restraint stress on the levels of MDA with and without SMF (0.8 mT) during 30, 60, and 240 min. The results were analyzed by analysis of variance (ANOVA) and Dunnett's multiple comparison test was used to compare the outcomes between the experimental and control groups. Data show the mean ± standard error (*n* = 8 per group). ^*∗∗∗*^*p* < 0.001 versus control group; ^###^*p* < 0.001 versus 240 min without SMF; °°°*p* < 0.001 and °*p* < 0.05 versus control group.

The MDA levels were significantly increased after the restraint stress in all experimental groups (30 min, 81%; 60 min, 85%; and 240 min, 97%) compared with the control group. Exposure to SMF for 240 min significantly decreased the MDA levels (40%), compared with the groups not exposed to SMF (Figure 7).

## 4. Discussion

Owing to the frequent exposure of living beings to magnetic fields, the sensitivity of different biological systems to magnetic fields has been studied for many years [41]. It is necessary to understand the influence of magnetic fields from different sources on the human body.

Magnetic fields are classified as weak (<1 mT), moderate (1 mT to 1 T), strong (1–5 T), and ultrastrong (>5 T) [22, 42, 43]. Here, we used a 0.8 mT magnetic field that corresponds to a weak source and to which humans are most frequently exposed.

It has been reported that SMFs are time-independent [44]. Hashish et al. [45] proposed four important parameters for assessing the interaction of SMFs with biological systems, including the target tissue, magnet characteristics, magnet device support, and dosing regimen. SMFs are difficult to shield and can freely penetrate biological tissues [44]. However, apart from the field intensity, the gradient of the field also has an important role in the biological effects of SMFs [46].

The SMFs can interact directly with moving charges (ions, proteins, and others), as well as with magnetic materials found in tissues, through several physical mechanisms [47, 48]. These include the production of ROS by triplet-singlet modulation of semiquinone flavin (FADH) enzymes, which are O_2_ spin-correlated radical pairs, thus resulting in flavin decoupling and an increase of H_2_O_2_, inducing as such cellular oxidative stress [48, 49]. Indeed, several reports have shown that these processes modulate the endogenous and exogenous production of ROS [50].

In the present study, we demonstrated an oxidant-antioxidant imbalance after acute immobilization stress and exposure to SMF for different periods. We also observed an increase in plasma NO levels with acute immobilization stress, which is consistent with previous studies [51, 52]. The immobilization/restraint stress is a well-known method for producing chronic and acute stress [21] and mimics the natural stress progression by inducing both physiological and physical stress [20]. So the increase of NO, which interacts with superoxide anions (O_2_^−^) and thiol compounds, generates reactive nitrogen species (RNS), peroxynitrite, and S-nitrosothiol [53, 54]. Likewise, an increase in NO has been reported after exposure to other sources as well [55].

At the same time, mitochondrial oxidative phosphorylation is the major intracellular source of reactive oxygen species (ROS): superoxide, peroxide, and hydroxyl radicals; produced in the electron transport chain [56]; Iorio et al. reported that Low-frequency magnetic fields (LFMFs), with a square waveform of 5 mT amplitude and frequency of 50 Hz, could increase energy generation through regulating mitochondrial oxidative phosphorylation [57] assuming that LFMFs suppress ROS through regulating mitochondria function.

Another study shows that ROS generation (including O_2_^−^ and ONOO^−^) and NO production were suppressed combined with decreased NADPH oxidase activity when exposed to LFMFs in a model of cardiac ischemia reperfusion [58].

In this experiment, we showed that the NO levels decreased after exposure to SMF at all three exposure times may be used, but most severely after exposure for 240 min. Other studies have reported that exposure to extremely low-frequency magnetic fields does not increase the NO production [59, 60].

Cells are known to use an antioxidant defense system to scavenge ROS to alleviate the cumulative burden of oxidative stress. SOD and GSH form the first line of defense against oxidative stress and can inhibit free-radical formation and prevent oxidative damage [61, 62].

Superoxide dismutase, or SOD, is an enzyme that plays an important role in O_2_^−^ metabolism, preempting the oxidizing chain reaction that causes extensive damage and forestalling the formation of a cascade of deleterious reactive ROS; the level of SOD was also therefore determined in this study.

It has been reported that the restraint stress per se increases the O_2_^−^ content. Exposure to SMF (0.8 mT for 60 and 240 min) increased the SOD activity significantly compared with the restraint groups. The activity of SOD, which decreases the O_2_^−^ levels, accelerated its conversion to hydrogen peroxide (H_2_O_2_). In addition, O_2_^−^ and H_2_O_2_ react with each other (Heber-Weiss reaction), generating the hydroxyl radical (OH^−^), which attacks any target, including lipids, proteins, all DNA components, and many amino acids [62], so that the exposure to SMF distressing the redox balance leads to a decrease in the oxidative stress in the system.

GSH plays a crucial role by protecting the cell from endogenous ROS and RNS [63, 64]. Although it directly quenches free radicals, it may have a greater importance for its direct effect on oxidative stress. GSH has been shown to scavenge directly diverse oxidants such as O_2_^−^, OH^−^, nitric oxide, and carbon radicals. It also catalytically detoxifies hydroperoxides, peroxynitrites, and lipid peroxides [63]. In our results, decreased GSH plasma levels in rats that were exposed to acute immobilization stress and exposure to SMF for different periods significantly increased the levels of GSH, suggesting that the changes are dependent on the time of exposure and intensity field.

In addition, the SOD activity and GSH, which metabolizes O_2_^−^ and accelerates its conversion to H_2_O_2_, which is subsequently reduced to water by the GSH cycle, might also have contributed to damage reduction. Likewise, the depletion of H_2_O_2_ has an inhibitory effect on OH^−^ formation and decreases the lipid peroxidation values. AGEs are a heterogeneous group of compounds derived from no-enzymatic glycation proteins, lipids, and nucleic acids through complex and sequential reactions known as the Maillard reactions. Edeas et al. [39] have noted the importance of oxidizing conditions and ROS for the formation of AGEs.

Additionally, the AOPPs are widely used in oxidative stress research as protein oxidative markers [38].

The measurement of AGEs and AOPPs revealed that highly reactive aldehydes (-CHO) or dicarbonyl group components (-CO-CHO) [64] could form through the autoxidation of sugars and lipid peroxidation. Furthermore, AOPPs are proteins (albumin), and their aggregates are often damaged by oxidative stress [38]. They mostly comprise dityrosines, which allow crosslinking with disulfides through chlorinated oxidants, hypochloric acid, and chloramines, resulting in myeloperoxidase activity [65, 66] and the formation of carbonyl groups [67]. These cause damage to important biological structures, such as proteins, carbohydrates, lipids, and nucleic acids, and may enhance the inflammatory response via NADPH oxidase [65] or myeloperoxidase action [66]. Thus, we showed that stress immobilization increases AOPP and AGEs levels and that exposure to SMF decreases.

There are authors who point out that, in fibrillogenesis processes, registered in populations of young and old, the collagen fibers are better aligned if subjected to magnetic fields and also AGES measurement behaves in this way. On the other hand, if the AGES are measured without subjecting them to magnetic fields, the content is high [68] without being exposed to a magnetic field.

A proposed mechanism, which is the acceleration of this process, can occur through the reaction of copper ions with H_2_O_2_ to generate free radicals and ROS [69] that react very rapidly with glucose. The result is the formation of much more reactive dicarbonyls compounds, which are associated with different pathology [70].

One possibility of explaining this effect is that the magnetic field deflected the charges that are moving and thereby contribute to these charges are coupled with others so that they neutralize, contributing to reduce oxidative stress.

MDA is one of the most frequently used indicators of lipid peroxidation in biomedical research owing to its high lability. In particular, linoleic acid, arachidonic acid, and OH^−^ and peroxyl radicals are important targets of lipid peroxidation of ROS, which cause autocatalytic chain reactions of lipid peroxidation [40, 70].

Previous studies have evaluated the influence of immobilization stress in the plasma and tissues of rat and reported lipid peroxidation and damage [71–74]. Exposure to SMF has shown interesting results. Ciejka et al. [74] showed that exposing rats to a magnetic field for 30 min every day for 10 days affected the lipid peroxidation reactions; however, when the exposure time was prolonged to 60 min/day, they found a decrease in lipid peroxidation and an increase in the content of -SH groups. This was likely due to an increase in the total thiol groups, which decreases oxidative stress and protects the tissues [38, 67]. Other studies have also reported similar results [65, 66]. We observed that stress increases the lipoperoxidation values, which showed a time-dependent response to SMF exposure. Therefore, our results were consistent with those of Wilson et al. [67, 75], who proposed an adaptive response to the activation by the SMF antioxidant system.

Some oxidative products (reactive aldehydes such as methylglyoxal) or lipid peroxidation products (e.g., MDA) may bind to proteins and amplify glycoxidation generated lesions. As a result, alteration of the cellular functionality, simultaneous stimulation of cytokine production, and increased inflammation and some acute phase reactants might consequently occur, thus accelerating different pathologies.

In summary, the reported biomarkers could contribute to changes in important biological structures (proteins, carbohydrates, lipids, and nucleic acids) in the immobilization stress model. A comprehensive description of the responses of oxidative stress biomarkers after a redox treatment would facilitate the establishment of biomarker sets, including only the most suitable biomarkers for the pathology under study.

## 5. Conclusions

In the present study, we proposed that exposure to weak SMFs could open new perspectives in health research, especially with regard to oxidative stress.
